# Coexpression network analysis reveals an MYB transcriptional activator involved in capsaicinoid biosynthesis in hot peppers

**DOI:** 10.1038/s41438-020-00381-2

**Published:** 2020-10-01

**Authors:** Binmei Sun, Xin Zhou, Changming Chen, Chengjie Chen, Kunhao Chen, Muxi Chen, Shaoqun Liu, Guoju Chen, Bihao Cao, Fanrong Cao, Jianjun Lei, Zhangsheng Zhu

**Affiliations:** 1 Key Laboratory of Biology and Genetic Improvement of Horticultural Crops (South China), Ministry of Agriculture and Rural Affairs, College of Horticulture, South China Agricultural University, Guangzhou, 510642 China; 2 Jiangxi Agricultural Engineering College, Zhangshu, 331200 Jiangxi China; 3 Guangdong Helinong Seeds, Co., Ltd., Shantou, 515800 Guangdong China; 4 Guangdong Helinong Agricultural Research Institute, Co., Ltd., Shantou, 515800 Guangdong China; 5 Henry School of Agricultural Science and Engineering, Shaoguan University, Guangdong, 512005 China; 6 Peking University—Southern University of Science and Technology Joint Institute of Plant and Food Sciences, Department of Biology, Southern University of Science and Technology, Shenzhen, 518055 China

**Keywords:** Secondary metabolism, Plant physiology

## Abstract

Plant biosynthesis involves numerous specialized metabolites with diverse chemical natures and biological activities. The biosynthesis of metabolites often exclusively occurs in response to tissue-specific combinatorial developmental cues that are controlled at the transcriptional level. Capsaicinoids are a group of specialized metabolites that confer a pungent flavor to pepper fruits. Capsaicinoid biosynthesis occurs in the fruit placenta and combines its developmental cues. Although the capsaicinoid biosynthetic pathway has been largely characterized, the regulatory mechanisms that control capsaicinoid metabolism have not been fully elucidated. In this study, we combined fruit placenta transcriptome data with weighted gene coexpression network analysis (WGCNA) to generate coexpression networks. A capsaicinoid-related gene module was identified in which the MYB transcription factor CaMYB48 plays a critical role in regulating capsaicinoid in pepper. Capsaicinoid biosynthetic gene (CBG) and CaMYB48 expression primarily occurs in the placenta and is consistent with capsaicinoid biosynthesis. CaMYB48 encodes a nucleus-localized protein that primarily functions as a transcriptional activator through its C-terminal activation motif. CaMYB48 regulates capsaicinoid biosynthesis by directly regulating the expression of CBGs, including *AT3a* and *KasIa*. Taken together, the results of this study indicate ways to generate robust networks optimized for the mining of CBG-related regulators, establishing a foundation for future research elucidating capsaicinoid regulation.

## Introduction

Hot pepper (*Capsicum* spp.) is the most popular vegetable and ingredient in the world because of its attractive spicy flavor^1,2^. The pungent taste originates from a group of alkaloid compounds, capsaicinoids, which are uniquely produced by the *Capsicum* genus^2^. To date, more than 22 capsaicinoids have been identified in hot pepper; capsaicinoid (Cap) and dihydrocapsaicin (DhCap) are the two major components and account for ~90% of the total capsaicinoid content^3–5^. Capsaicinoids are exclusively synthesized in the pepper placenta, mainly from 16 days post-anthesis (DPA) to the mature green stage through the condensation reaction of the precursors derived from phenylpropanoid and the branched-chain fatty acid pathway^6–8^. Capsaicinoid biosynthetic genes (CBGs), such as *CCoAMT*, *AMT*, *BCAT*, *KasIa*, *ACL*, *KR*, *FatA*, and *AT3a*, have been identified as being involved in capsaicinoid biosynthesis^3,8–13^. In nature, pepper synthesizes capsaicinoids for antifungal and antibacterial activities, and these compounds also act as a deterrent to mammals^14^. For humans, capsaicinoids function as bioactive compounds because of their many benefits: they are not only effective in treating many diseases, such as cancer and obesity, as well as pain^15,16^, but are also added to food for sterilization^17^ and widely applied in riot control and personal defense spray agents^18^. Since capsaicinoids have considerable application value and commercial purposes, several methods have been employed to increase the capsaicinoid content^5,19,20^. However, the capsaicinoid biosynthesis process is strictly switched spatially and temporally, and the expression of CBGs in pepper is precisely regulated at the transcriptional level^4,21^. Altering the expression of key CBGs in pepper seems to be effective in improving capsaicinoid contents. In highly pungent peppers, the transcriptional level of CBGs (e.g., *AMT*, *KasIa*, and *AT3a*) is consistently higher than that in weakly pungent cultivars^3,5,21^. Remarkably, the expression level of a major transcription factor usually affects the transcript level of all genes in the biosynthetic pathway, which will ultimately change the accumulation of compounds^5,22,23^. For example, *Catharanthus roseus ORCA3* is a crucial transcription factor that regulates the expression of terpenoid indole alkaloid genes, and overexpression of *ORCA3* results in 3.2-fold-increased accumulation of terpenoid indole alkaloids^24^. Similarly, in horticultural plants, such as tomato (*Solanum lycopersicum*)^25^, tea (*Camellia sinensis*)^26^, and orange (*Citrus sinensis*)^27^, high accumulation of health-promoting anthocyanins, which arise from upregulation of the anthocyanin-related MYB transcriptional activator, leads to anthocyanin biosynthetic gene expression simultaneously. Although the Solanaceae-specific MYB transcription factor *MYB31* was determined to be involved in capsaicinoid biosynthesis^5,28^, the transcriptional regulation of capsaicinoid biosynthesis has not been fully elucidated. Therefore, the identification of capsaicinoid biosynthesis-related transcription factors is needed.

A number of research efforts have focused on the biosynthesis of capsaicinoids to facilitate manipulations of pepper capsaicinoid content. Previously, the identification of genes relevant to capsaicinoid biosynthesis was performed via a combination of genetic fine mapping and reverse genetic and biochemistry approaches^3,8^. However, this method was hindered by the construction of a mapping population, which is laborious, and genetic transformation is highly difficult. In subsequent research, considerable progress has been achieved in RNA sequencing (RNA-seq). Identification of capsaicinoid biosynthesis candidate genes through transcriptomic data appears to be a more efficient strategy^4,11^. Gene coexpression network analysis (GCNA) is a systems biology approach for describing the correlation patterns between genes across large-scale gene expression profiling data^29^. To date, GCNA has been successfully applied in various biological contexts. In particular, these studies have provided key insights into plant secondary metabolism processes^30^. For instance, GCNA was used as a valid approach to identify biosynthetic genes from mayapple that complete the biosynthetic pathway to the etoposide aglycone^31^. In parallel, this technique also highlighted the role of GAME9 in regulating steroidal alkaloid biosynthesis in tomato and potato^32^. As one of the GCNA-based approaches, weighted GCNA (WGCNA) is a correlation-based technique that describes and visualizes coexpression networks between genes using transcriptomic data^33^. Currently, WGCNA is the most popular method used to identify and dissect gene modules in specific biological processes in a variety of plant species^34^. Therefore, utilization of WGCNA will facilitate the identification of novel potential regulators related to capsaicinoid biosynthesis. Despite these enlightening reports, little is known about the use of WGCNA to identify transcription factors involved in the regulation of capsaicinoid metabolism.

Capsaicinoid biosynthesis exclusively occurs in the pepper fruit placenta and in response to combinatorial developmental cues that are controlled at the transcriptional level. The availability of a large amount of RNA-seq data in pepper provides an ideal approach for investigating the regulation of capsaicinoid biosynthesis. In this study, through WGCNA, we identified a module related to capsaicinoid. In this module, we identified and characterized an R2R3 MYB transcription factor, CaMYB48, involved in capsaicinoid biosynthesis. CaMYB48 positively regulates capsaicinoid accumulation in pepper by directly activating CBGs. This study describes ways to generate robust networks optimized for the identification of CBG-related regulators, establishing a foundation for further research investigating the regulatory network of capsaicinoids.

## Results

### Identification of the capsaicinoid-related module

**Fig. 1:**
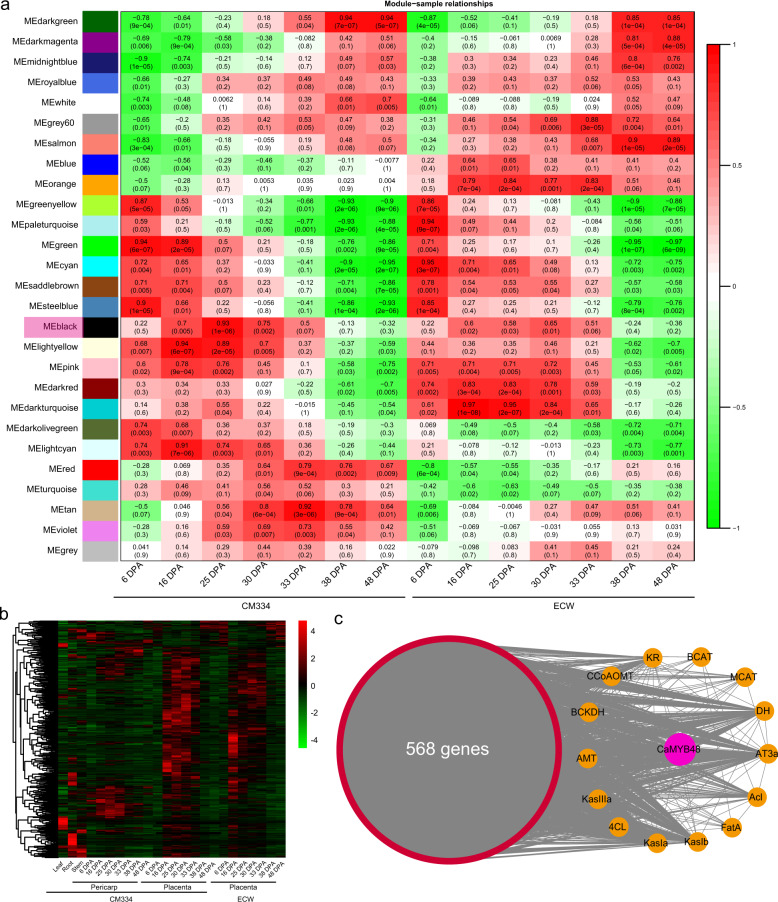
WGCNA of the pepper placenta at different developmental stages. **a** Module-tissue association. Each row corresponds to a module. Each column corresponds to the cultivar CM334 and ECW fruit placenta at a certain developmental stage. The color of each cell at the row–column intersection indicates the correlation coefficient between the module and the placenta developmental stage. A high degree of correlation between a specific module and the placenta developmental stage is indicated by red. The module with the highest correlation between capsaicinoid biosynthesis and gene expression is indicated by the red box covering the module name. **b** Heat map of genes in the MEblack module. Gene expression-level data in different tissues of cultivar CM334 and ECW. **c** Network analysis of the CBGs and *CaMYB48* in the MEblack module

Capsaicinoid biosynthesis primarily occurs in the pepper fruit placenta. Capsaicinoid levels are highly dynamic during fruit development and appear to be influenced by the ontogenetic trajectory of the fruit^4,5^. Capsaicinoids begin to accumulate at the fruit developmental stage of 16 DPA and peak at the mature stage (~40 DPA). Accordingly, key CBGs (i.e., *AMT, KasIa*, and *AT3a*) are also highly expressed in the placenta from 16 DPA to the mature green stage (approximately 30 DPA) during pepper placenta development. This highly dynamic process is precisely governed at the transcriptional level. To understand the landscape of transcription regulation of capsaicinoid biosynthesis, transcriptomic data of the pungent cultivar CM334 (*Capsicum annuum*) and nonpungent cultivar ECW (*C. annuum*) placenta at seven different developmental stages were retrieved from previous studies^4^. Coexpression networks were generated via the WGCNA package, and the combinational 14-placenta-development-stage network incorporated 26 clusters of coexpressed genes (Fig. 1a and Supplementary Fig. 1). The eigengenes were recognized as the first principal component of a cluster and can be thought of as representative of a cluster’s expression profile. The expression level of each cluster’s eigengene in each of the placenta tissues is plotted in a heat map (Fig. 1a), which enables easy visualization of the cluster placenta developmental stage association. Capsaicinoid biosynthesis occurs from 16 DAP to 30 DPA (mature green stage), and many CBGs are also highly expressed during this stage. Among the 26 distinct modules, the MEblack module had an expression pattern tightly correlated with capsaicinoid biosynthesis (Fig. 1b and Supplementary Figs. 2, 3). We found that in both CM334 and ECW, the genes in cluster MEblack were expressed most highly from 16 DPA to 30 DPA. In addition, the MEblack genes of different tissues were plotted in a heat map, and most of these genes exhibited a placenta-preferred or placenta-specific pattern (Fig. 1b). In total, 568 genes were included in this module: 14 CBGs, including key CBGs, such as *AMT*, *KasIa*, and *AT3a* (Fig. 1c and Supplementary Table 1). In addition, 20 out of 568 genes were identified as transcription factors (Supplementary Table 2). Notably, among these transcription factors was an MYB transcription factor, *CA11g12490*, referred to in this study as *CaMYB48*, which was tightly associated with the MEblack module (kME *P* value = 1.42E − 08). Moreover, *CaMYB48* was found to be highly coexpressed with CBGs. Since a vast number of MYB transcription factors have been characterized as being involved in regulating plant specialized metabolite biosynthesis^35^, we speculate that CaMYB48 might play an important role in regulating capsaicinoid biosynthesis.

### *CaMYB48* and CBG expression patterns are spatiotemporally specific

**Fig. 2:**
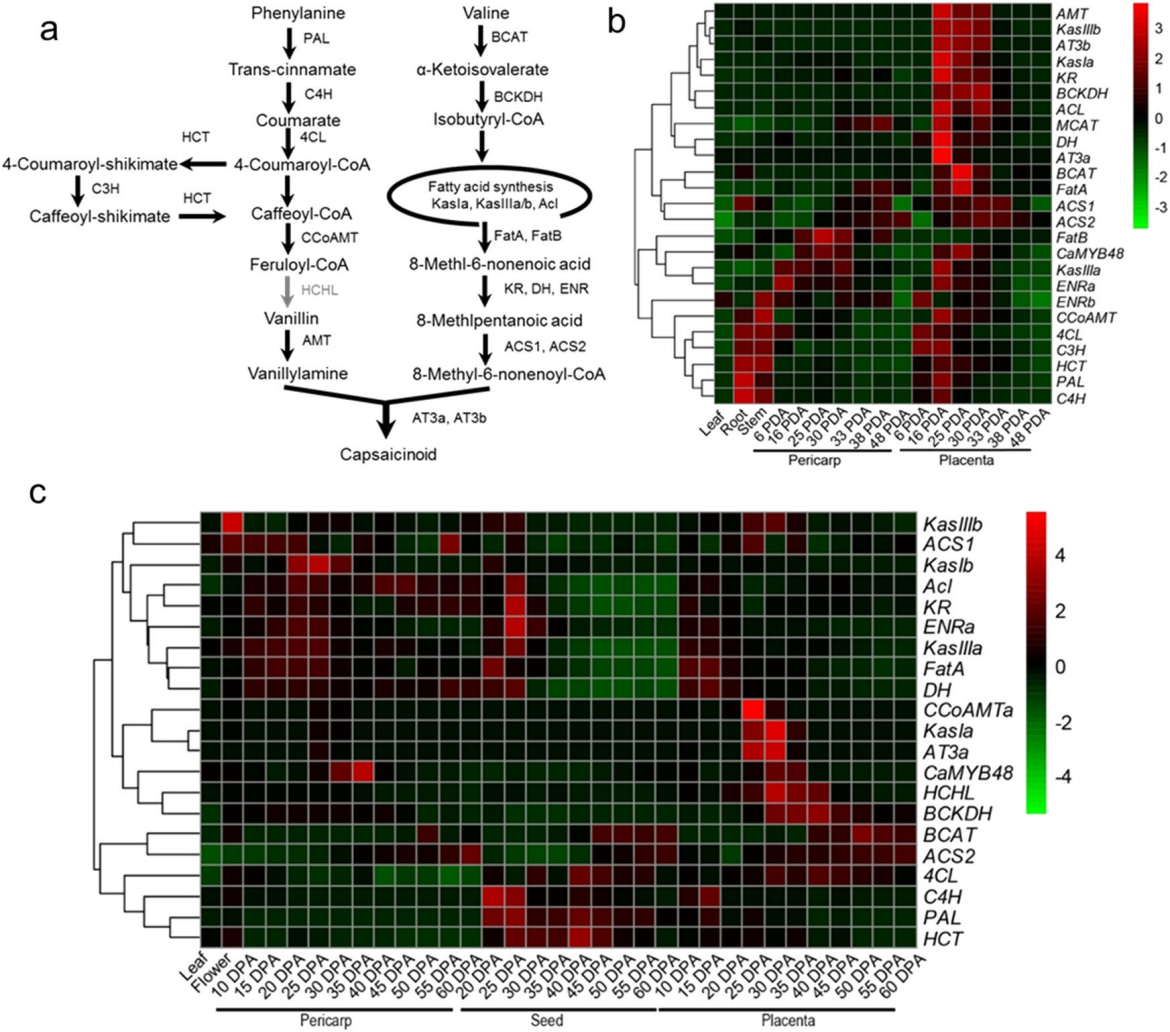
Expression analysis of CBGs and CaMYB48. **a** Capsaicinoid biosynthetic pathway. Some CBGs involved in capsaicinoid biosynthesis are not presented in the biosynthetic pathway schematic diagram. **b** Heat map showing CBG and *CaMYB48* gene expression in pepper cultivar CM334. **c** Heat map of CBG and *CaMYB48* gene expression in the 6421 inbred line

To better understand the relationship between *CaMYB48* and capsaicinoid biosynthesis pathway gene expression (Fig. 2a), different sets of raw pepper transcriptome data were retrieved from previous studies^4,36^. The results indicated that the expression of CBGs in pungent cultivar CM334 was gene-dependent (Fig. 2b). The upstream biosynthetic pathway genes (e.g., *PAL* and *C4H*) did not display tissue-specific expression patterns. In contrast, most downstream CBGs exhibited a spatiotemporally specific expression pattern, i.e., high expression in the 16–30 DPA placenta. Notably, a high expression level of *CaMYB48* was also detected at 16–30 DPA in the placenta (Fig. 2b). However, a considerable expression level of *CaMYB48* was also detected in the pericarp during this stage, indicating that *CaMYB48* may also have certain functions in the pericarp. To further reveal whether *CaMYB48* expression and CBG expression exhibit similar patterns among different cultivars, transcriptome data of the 6421 pungent inbred line at different fruit developmental stages (11 stages) were also retrieved from previous studies^36^. Consistent with the results observed in CM334, *CaMYB48* was highly coexpressed with downstream CBGs. Taken together, these results suggested that CaMYB48 might regulate the coexpressed CBGs to regulate capsaicinoid biosynthesis.

### CaMYB48 is associated with capsaicinoid biosynthesis

**Fig. 3:**
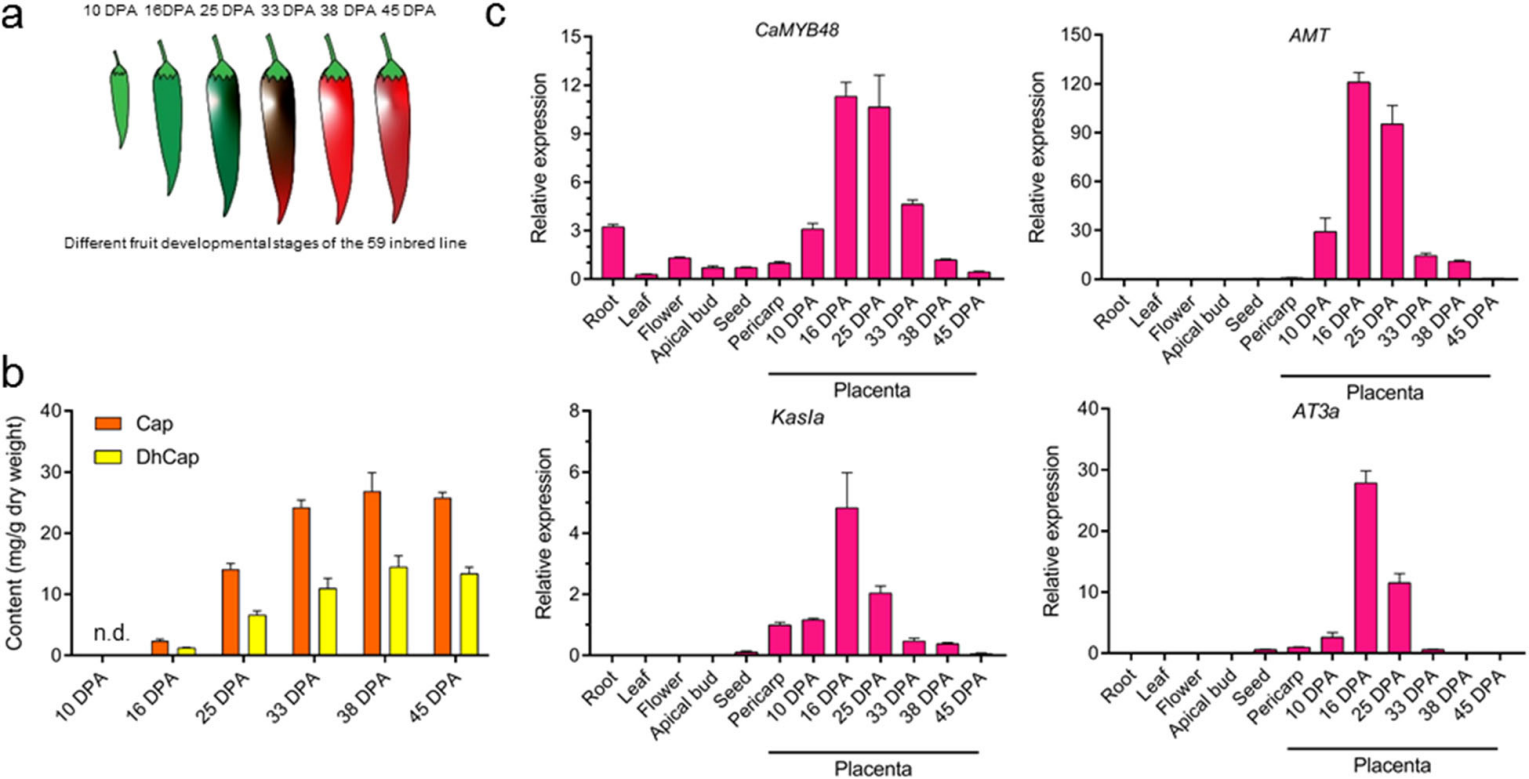
*CaMYB48* expression is associated with capsaicinoid biosynthesis. **a** Schematic diagram of pepper fruit at different developmental stages. **b** The contents of Cap and DhCap in the fruit placenta at different developmental stages. The fruit placenta of the 59 inbred line at different stages was used for analysis. n.d. indicates undetectable. Data are expressed as the mean ± SD (*n* = 3). **c** Transcript levels of *CaMYB48* and CBGs in different tissues. Roots, leaves, stems, and apical buds were collected from 30-day-old seedlings. Fully blossomed flowers were collected from 80-day-old plants. Seeds and pericarp were collected from 16-DPA fruit, and the placenta was collected at different fruit developmental stages. The relative expression of the pericarp was set to 1, and that of all the other tissues was measured relative to that of the pericarp. Data are expressed as the mean ± SD (*n* = 3)

To characterize the capsaicinoid contents in the fruit placenta of elite inbred line 59 (*C. annuum*) during different developmental stages, we adopted high-performance liquid chromatography (HPLC) to analyze extracts from six developmental stages (10, 16, 25, 33, 38, and 45 DPA) (Fig. 3a). The abundances of two major capsaicinoids, Cap and DhCap, gradually increased during development, with a rapid increase from 16 DPA to 33 DPA and a peak at 38 DPA (Fig. 3b). Real-time quantitative reverse transcription PCR (qRT-PCR) analysis was performed to dissect the relationship between *CaMYB48* and capsaicinoid biosynthesis. The expression pattern of *CaMYB48* and CBGs was displayed in a tissue- and development-dependent manner. The results revealed a high abundance of *CaMYB48* detected in the placenta from 16 DPA to 33 DPA, and the key CBGs *AMT, KasIa*, and *AT3a* were also highly expressed at these stages (Fig. 3c), implying that CaMYB48 might govern the expression of these genes. However, we found that *CaMYB48* also has considerable expression levels in other tissues, such as roots, pericarps, and flowers, indicating that it functions in these tissues. Taken together, these results strongly indicate that CaMYB48 is a candidate regulator of capsaicinoid biosynthesis.

### *CaMYB48* encodes a nucleus-localized R2R3 MYB transcription factor

**Fig. 4:**
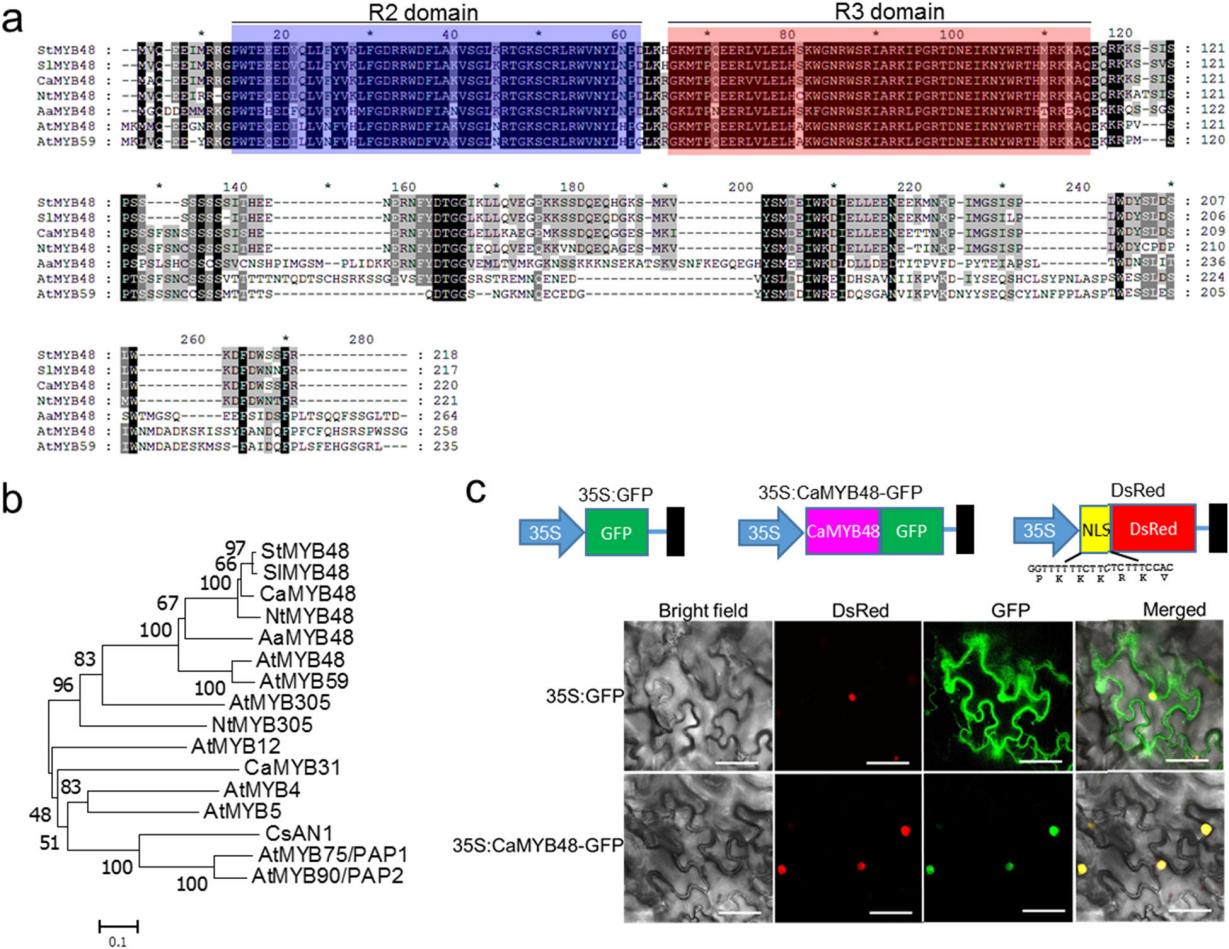
Phylogenetic and subcellular analyses of CaMYB48. **a** Sequence analysis of CaMYB48 and other MYB transcription factors. To compare the MYB sequences among different plant species, CaMYB48 was used as a bait gene, and MYB transcription factors sharing highly similar sequences in *Solanum lycopersicum, Solanum tuberosum, Nicotiana tabacum, Artemisia annua and Arabidopsis thaliana* were retrieved from the NCBI database. **b** Phylogenetic tree of MYB transcription factors. Some MYB transcription factors involved in secondary metabolism were also used for phylogenetic analysis. A phylogenetic tree was constructed by MEGA7 using the neighbor-joining method with 1000 bootstraps. **c** CaMYB48 localized in the nucleus. *Agrobacterium tumefaciens* harboring 35S:GFP (empty control) or 35S:CaMYB48-GFP was infected into *N. benthamiana*. The nucleus-localized signal (NLS) fused to DsRed served as a nuclear marker. *Agrobacterium tumefaciens* harboring DsRed protein was also infected into *N. benthamiana*. Bar = 50 μm

The full-length coding sequence of *CaMYB48* was cloned from the placenta of the 59 inbred line. Nucleotide sequence analysis indicated that the coding sequence of *CaMYB48* was 669 bp in length, and the deduced amino acid sequence was 222 amino acids (Fig. 4a). The MYB transcription factors that shared the highest sequence identity with CaMYB48 were retrieved from *Solanum lycopersicum*, *Solanum tuberosum*, *Nicotiana tabacum*, *Artemisia annua*, and *Arabidopsis thaliana*. Sequence analysis indicated that the protein contained R2 and R3 MYB domains within the N-terminus (Fig. 4a), which play an important role in DNA binding. To dissect the potential function of CaMYB48, phylogenetic analysis was performed. The MYB transcription factors that shared the highest sequence identity with CaMYB48 from other plant species were retrieved from public databases, while some functionally identified MYB transcription factors were also adopted for analysis. As shown in the phylogenetic tree, within the CaMYB48 clade, the nearest MYB transcription factors from Solanaceae plants, i.e., *SlMYB48* (*Solanum lycopersicum*), *StMYB48* (*Solanum tuberosum*), and *NtMYB48* (*Nicotiana tabacum*), shared a sequence identity of more than 90% (Fig. 4b). However, the MYB functions in these Solanaceae plants are unknown. The most closely related MYB transcription factor from *Arabidopsis thaliana* was AtMYB48, which showed only a 53% amino acid sequence identity, and its function was not characterized.

Transcription factors execute transcriptional regulation depending on localization in the nucleus. To investigate the subcellular localization of CaMYB48, the coding sequence of CaMYB48 was fused in frame with the GFP gene to generate a 35S:CaMYB48-GFP construct (Fig. 4c). The SV40 nucleus-localized signal (NLS) fused with the DsRed protein to generate the nuclear marker. 35S:GFP or 35S:CaMYB48-GFP was coexpressed with DsRed in *Nicotiana benthamiana* leaf epidermal cells. Using confocal microscopy, we observed that the CaMYB48-GFP fusion protein colocalized with DsRed (Fig. 4c), confirming that CaMYB48 functions as a nucleus-localized transcription regulator. In contrast, in the empty vector, the GFP signal was observed in both the cytoplasm and nucleus of the epidermal cell.

### CaMYB48 functions as a transcriptional activator

**Fig. 5:**
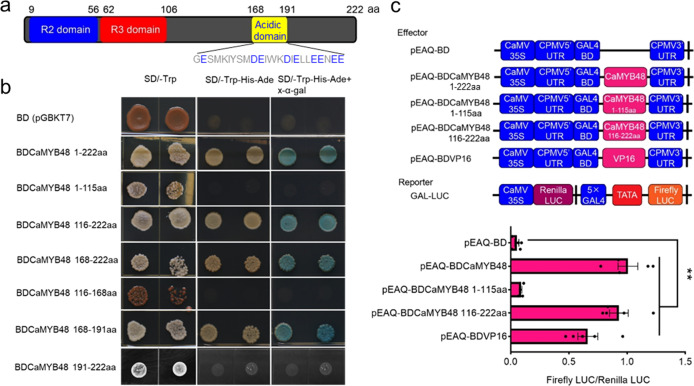
CaMYB48 is a transcription activator. **a** Schematic diagram of the CaMYB48 domain. Within the acidic domain, the acidic amino acids are marked in blue. **b** Activation analysis of CaMYB48 in yeast. Full-length and truncated CaMYB48 was used for activation analysis. The number shown on the right indicates the protein region used for activation analysis. Auxotroph plates of SD/–Leu–His–Ade (middle) and SD/–Leu–His–Ade–x-α-gal (right) showing transcriptional activation of protein. **c** CaMYB48 process transcriptional activation *in planta*. The number shown on the right indicates the protein region used for analysis in *N. benthamiana* leaves. The transcriptional activator VP16 was used as the positive control. Data represent the mean ± SD (*n* = 5). Student’s *t* test was used to identify significant differences compared to the empty vector control (**P < 0.01)

To identify the potential conserved motif in CaMYB48, the amino acid sequence was searched via the Multiple Em for Motif Elicitation (MEME) database. In addition to the R2 and R3 MYB repeats in the N-terminus, an acidic domain was also observed in the C-terminus (Fig. 5a). To determine how these motifs associate with CaMYB48 transactivation ability, we performed a transactivation activity assay in yeast and plants to test the transcriptional activation activity of CaMYB48. Based on the presence of conserved domains, full-length or truncated CaMYB48 was fused in frame with the GAL4 DNA-binding domain (BD) in the pGBKT7 (BD) vector, and the constructs were transformed into the Y2H Gold yeast strain. The growth of yeast carrying BDCaMYB48 on SD/−Trp/−His/−Ade selective medium along with an x-α-gal assay indicated that the CaMYB48 protein has strong activation activity (Fig. 5b). To determine how the structure of CaMYB48 influences its activation activity, the CaMYB48 protein was divided into two major parts based on conserved domain analysis: the R2R3 repeat domain (1–115 aa) and the C-terminal domain (116–222 aa). Analyzing truncated CaMYB48 in the yeast assay indicated that aa 168–191 were essential for the activation activity of CaMYB48 (Fig. 5b). To explore whether this transcriptional activation ability is mediated in plants, a dual-luciferase (LUC) reporter assay was performed. The full-length or truncated *CaMYB48* was fused in frame with the GAL4 BD to generate an effector (Fig. 5c). The effector and reporter were coexpressed in *Nicotiana benthamiana* leaves via *Agrobacterium*-mediated transformation. Compared with the empty control, CaMYB48 strongly enhanced the relative activity of firefly LUC, suggesting that CaMYB48 has transcriptional activity in *N. benthamiana* (Fig. 5c). Further analysis indicated that the C-terminal acidic amino acid region was responsible for executing the transcriptional activity of CaMYB48 (Fig. 5c). Taken together, these data indicate that CaMYB48 functions as a transcriptional activator and that the C-terminal acidic amino acid region is necessary and sufficient for its transcriptional activity in yeast and *N. benthamiana*.

### Downregulation of *CaMYB48* decreases CBG expression and capsaicinoid content

**Fig. 6:**
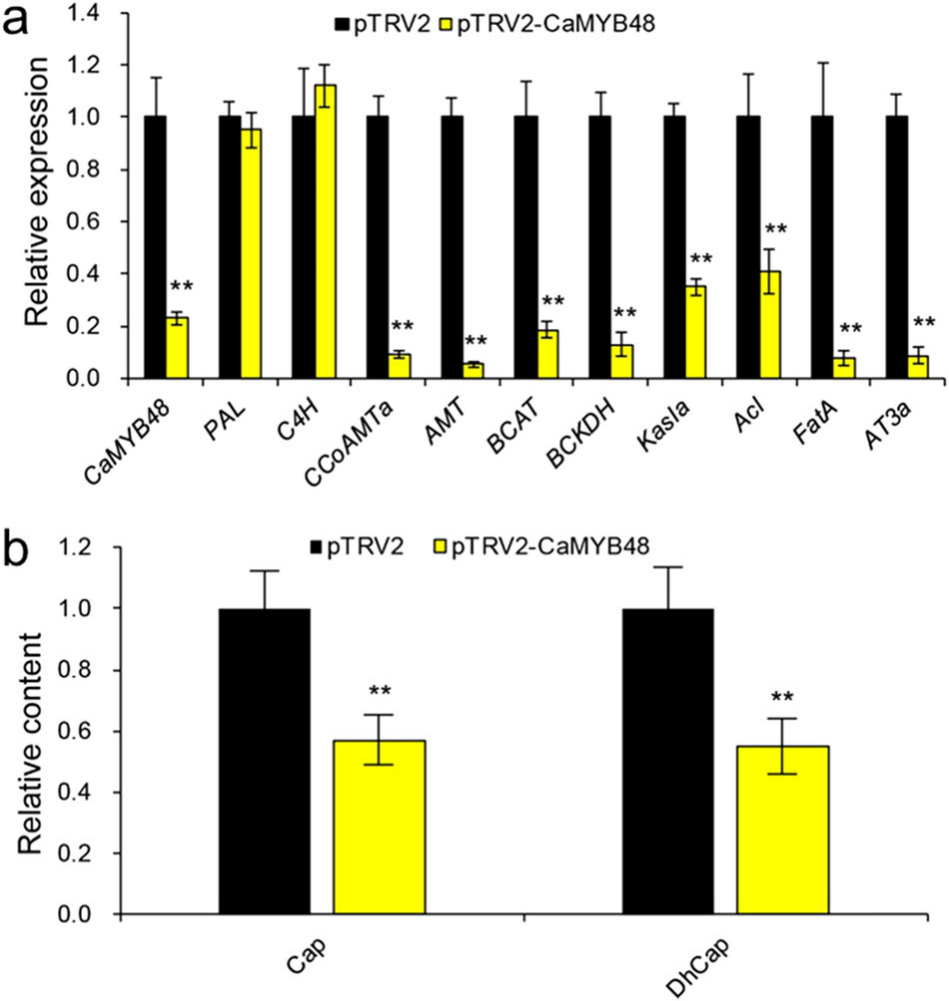
Silencing of MYB48 decreased the CBG transcription level and capsaicinoid content. **a** Relative expression of *CaMYB48* and CBGs in the placenta of the 59 inbred line at developmental stage 16 DPA in *CaMYB48*-silenced plants compared to control plants. Data represent the mean ± SD (*n* = 5). Student’s *t* test was used to identify significant differences compared to the control (**P < 0.01). **b** VIGS of *CaMYB48* decreased the Cap and DhCap contents. The placenta of the 59 inbred line was measured for capsaicinoid content at 45 DPA. Data represent the mean ± SD (*n* = 5)

To reveal the function of CaMYB48 in peppers, a loss-of-function analysis of *CaMYB48* via virus-induced gene silencing (VIGS) was carried out to silence *CaMYB48* in the 59 inbred line. The silencing efficiency was evaluated by qRT-PCR analysis of the *CaMYB48* transcription level in the placenta at the developmental stage of 16 DPA. Compared with that in the control, the *CaMYB48* transcription level in the *CaMYB48-*silenced plants was reduced threefold (Fig. 6a). The expression of the upstream CBG (e.g., *PAL* and *C4H*), which is derived from the phenylpropanoid pathway, changed slightly in both the control and CaMYB48-silenced plants. In contrast, the expression of the downstream CBG changed significantly, and the percentage of genes that decreased transcript levels ranged from ~60 to 90% (Fig. 6a). Consistent with the downregulation of CaMYB48 and CBGs in *CaMYB48*-silenced plants, the Cap and DhCap contents decreased by 43% and 45%, respectively, compared to those in the control plants (Fig. 6b). Most of the downregulated CBGs were consistent with the coexpression network genes identified in the MEblack module, strongly supporting that CaMYB48 regulates certain coexpressed CBGs to control capsaicinoid biosynthesis.

### CaMYB48 can directly bind to and activate the transcription of CBG promoters

**Fig. 7:**
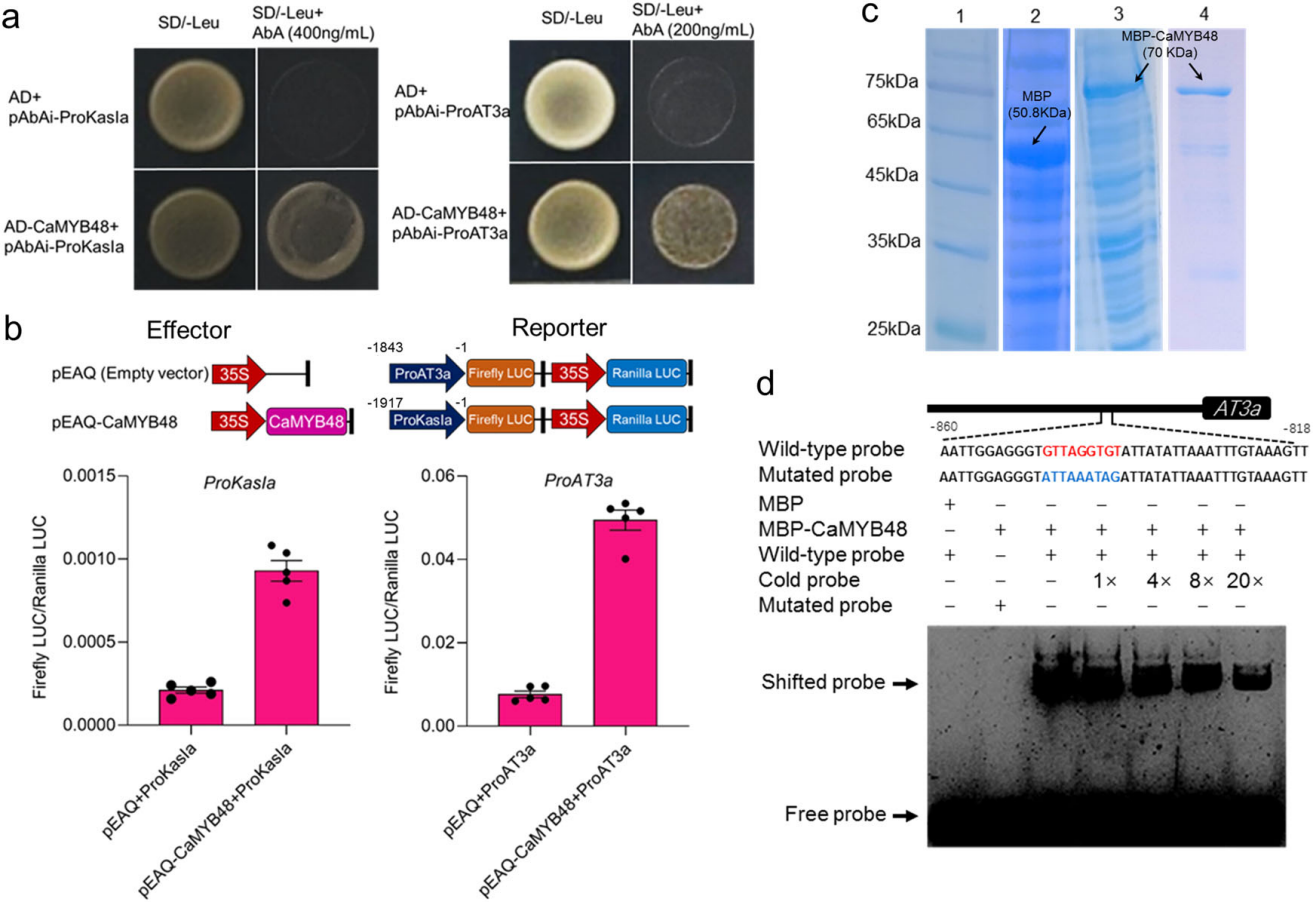
MYB48 targets the promoters of CBGs. **a** Y1H analysis of CaMYB48 associated with the promoters of *KasIa* and *AT3a*. SD/–Leu medium lacking leucine, AbA aureobasidin A. **b** CaMYB48 can activate *KasIa* and *AT3a* gene promoter transcription in *N. benthamiana* leaves. Data represent the mean ± SD (*n* = 5). **c** Expression and purification of CaMYB48. The protein expressed in *E. coli*. Lane 1, protein ladder; lane 2, pMAL-c2x empty vector; lane 3, pMAL-c2x vector recombinant with CaMYB48; lane 4, purified recombinant MBP-CaMYB48 protein. MBP maltose-binding protein. **d** EMAS of in vitro binding of the CaMYB48 protein to the promoter of *AT3a*. For competition, 1- to 20-fold excess unlabeled probes (cold probes) were mixed with biotin-labeled probes

Since *CaMYB48*-coexpressed CBGs were downregulated in *CaMYB48*-silenced plants, we hypothesized that these genes may be directly targeted by CaMYB48. Two key CBGs, *AT3* and *KasIa*, were selected to verify the hypothesis. To determine whether *AT3*a and *KasIa* are directly targeted by CaMYB48, we performed a yeast one-hybrid (Y1H) analysis. The promoters of *KasIa* and *AT3a* were ligated into the pAbAi vector to generate bait vectors (pAbAi-ProKasIa and pAbAi-ProAT3a). CaMYB48 was ligated into pGADT7 to generate a prey vector. Subsequently, the bait and prey vectors were transformed into the Y1H Gold yeast strain and screened on SD/–Leu medium containing a given concentration of aureobasidin A. The results indicated that CaMYB48 associates with the *KasIa* and *AT3a* promoters in yeast. The results also indicated that the Y1H Gold yeast strain cotransformed with the AD-CaMYB48 vector and that the promoters of *KasIa* and *AT3a* grew well on medium containing AbA (Fig. 7a). In contrast, the yeast cells cotransformed with empty vector, and the CBG promoters did not grow well on the AbA-containing medium. These results indicated that CaMYB48 is associated with the *AT3a* and *KasIa* promoters in yeast. To confirm whether the transcription of *KasIa* and the *AT3a* promoter can be activated by CaMYB48 in vivo, a dual-LUC reporter assay was performed. The upstream start codons of 1843 bp of *AT3a* and 1917 bp of *KasIa* were used for assessment. Analysis of relative firefly LUC activity showed that the *AT3a* and *KasIa* promoter transcription levels were strongly activated by CaMYB48 in *N. benthamiana* leaves (Fig. 7b). R2R3 MYB transcription factors are known for binding to the MYB *cis*-element of target genes to execute transcriptional regulation processes^37^. Therefore, we analyzed the *AT3a* promoter sequence and identified several MYB cis-elements. To further investigate whether CaMYB48 can directly bind to the MYB cis-elements located at −849 to −841 bp upstream of the *AT3a* start codon, EMSAs were carried out. To determine whether CaMYB48 can bind to the *AT3a* promoter MYB cis-element, an electrophoretic mobility shift assay (EMSA) was carried out. The full-length coding sequence of CaMYB48 was ligated into pMAL-c2X and transformed into BL21-competent *E. coli*. The recombinant maltose-binding protein (MBP) of CaMYB48 was expressed and purified with amylose resin (Fig. 7c). As the labeled probes were incubated with CaMYB48 recombinant proteins, the probes exhibited slowed bands, and the binding clearly decreased following incubation with unlabeled probes as competitors (Fig. 7d). Taken together, our results indicate that CaMYB48 targets the *KasIa* and *AT3a* promoters by directly binding to their promoter-binding cis-elements.

## Discussion

### Coexpression facilitates the identification of capsaicinoid-related modules

Plant biosynthesis of specialized metabolites is often limited to specific tissues or exclusively occurs in response to environmental stimuli^38^. Regulation of specialized metabolite biosynthesis seems to be controlled at the transcriptional level, which is generally dependent on the interaction of DNA-related mechanisms and the activity of transcription factors that may act in a combinatorial manner^35,38^. Capsaicinoids serve as defensive compounds to serve as deterrents against microorganisms and mammals to protect seeds from damage^4,5,21^. Pepper is a nonmodel horticultural crop, and the mining of biosynthetic genes and transcription regulators is still hindered^4^. Previously, the identification of the capsaicinoid biosynthesis-related genes *AT3a*, *KasIa*, *CCoMTa*, *AMT*, *KR*, and *MYB31* was mainly based on genetic mapping, VIGS, and histochemistry approaches^3,5^. Omics-based approaches have become available, facilitating research on nonmodel pepper plants. In this study, through WGCNA, we identified a MEblack module that exhibited a tight correlation of gene expression patterns with capsaicinoid biosynthesis (Fig. 1). Notably, a larger number of functionally characterized CBGs, such as *AMT*, *AT3*, *KasIa*, and *KR*, were identified in this module. Prior to this study, no coexpression network analysis-based strategy had been adopted to identify the CBGs and related transcriptional regulators. Compared with the biosynthesis pathways of other specialized metabolites, the capsaicinoid biosynthesis pathway is fairly complicated, since the production of capsaicinoids occurs via the condensation of precursors derived from phenylpropanoid and branched-chain fatty acid pathways. Based on bioinformatic and transcriptomic analyses, more than 50 genes were proposed to be involved in capsaicinoid biosynthesis. Via WGCNA, 14 CBGs were detected in the MEblack module, and most of them belonged to pathways downstream of biosynthetic genes. Presumably, the expression of most MEblack module CBGs exhibited tissue specificity and developmental stage regulation patterns (Fig. 1). Indeed, compared to the expression of MEblack module CBGs, the expression of some genes, such as *PAL*, *HCT*, and *ACS*, did not show apparent tissue specificity. We speculate that some of these coexpressed CBGs in the MEblack module encode key enzymes to determine the enzyme reaction rate. This property can be inferred from the results in which the capsaicinoid content was determined by the key CBGs, including *AMT*, *KasIa*, *Acl*, and *AT3a*, at the transcriptional level^3,5^. Coregulation has been observed for genes across multiple pathways of specialized metabolism, such as etoposide aglycone^31^, steroidal glycoalkaloids^39^, and cucurbitacin^40^. These tight gene-to-metabolite correlations were also reflective of predicted fluxes through the relevant pathways. To date, more than 22 capsaicinoids have been identified in hot pepper, but the genes responsible for the diversification of capsaicinoids have not been elucidated. The identified MEblack module with a tight gene-to-metabolite correlation will be a heavily studied topic in future research on the genes responsible for diversified capsaicinoids.

### CaMYB48 is a transcription factor involved in capsaicinoid biosynthesis

Most MYB family proteins function as transcription factors with varying numbers of MYB repeat domains, conferring their ability to bind to DNA. The MYB family has selectively expanded in plant species, particularly through the large family of R2R3 MYBs. R2R3 MYB transcription factors have been documented to regulate different classes of specialized metabolites in plants, such as phenylpropanoids^37^, betaine^41^, glucosinolates^42^, and carotenoids^43^. These MYB factors appear to function in the coordinated control of metabolic genes, since they display similar expression patterns^44^. In this study, through WGCNA, we identified an MYB transcription factor, CaMYB48, that functions as a transcriptional activator to regulate capsaicinoid biosynthesis. The nonpungent pepper cultivar ECW has a 2.5-kb deletion in *AT3a* that spans the region from the promoter to the first exon^4^. Expression analysis indicated that *AT3a* was highly expressed in the pungent pepper cultivar CM334 and was barely expressed in the nonpungent pepper cultivar ECW. However, *CaMYB48* was also highly expressed in cultivar ECW, and all other CBGs showed similar expression except for *AT3a*. These results indicate that the lack of pungency in ECW resulted from the loss of *AT3a* expression without changes in the expression of *CaMYB48* and other CBGs.

Although the sequence identity of CaMYB48 and its orthologs is highly conserved in Solanaceae plants, the functions of these orthologs in other Solanaceae plants have not been determined (Fig. 4). BLASTP analysis revealed that the MYB transcription factor from other plant species most closely related to CaMYB48 shared a fairly low sequence identity (<50% amino acid sequence identity). In particular, the C-terminus of the amino acid sequence exhibited highly variable features among these proteins (Fig. 4a), which are recognized as crucial for defining MYBs from different subgroups^37,45^. For example, the *Arabidopsis* MYB transcription factor most closely related to CaMYB48 is AtMYB48, which exhibits only 53% sequence similarity. In addition, it is worth noting that the expression of CaMYB48 orthologs in different species displayed distinct patterns. In this context, Solanaceae MYB48 and the nearest MYB from other species appear to be derived from different ancestors. Alternatively, the functional and expression orthologs of CaMYB48 in different genera of plants underwent divergent evolution during or after speciation from ancestral species. However, the function of CaMYB48 orthologs in different species needs further study. We previously performed genetic analysis to identify *MYB31* and functionally characterize its involvement in capsaicinoid biosynthesis through the regulation of structural gene biosynthesis^5^. Considering the complexity of capsaicinoid biosynthesis, the results of this study and prior results indicate that a variety of MYBs from different clades may be involved in capsaicinoid regulation in peppers. Whether additional cofactors interact with CaMYB48 to form regulatory complexes has not been determined.

In addition to the R2R3 DNA-binding domain, the C-terminus executes countless molecular functions that are absolutely crucial for biological function^37^. The Solanaceae-specific MYB transcription factor MYB31 has a Solanaceae-specific motif in the C-terminus (amino acids 213–234), and this motif performs its function as a transcriptional activation domain^5^. Unlike the highly conserved R2R3 DNA-binding domain, transcriptional activation domains seem to be intrinsically disordered, and low sequence conservation has made it challenging to identify the amino acid composition features that underlie their activity. In this study, characterization of the function of CaMYB48 as a transcriptional activator provides insight into this activation mechanism. Our results illustrated that the C-terminal at 168–191 aa, which is rich in acidic amino acids, is critical for the activation activity of CaMYB48. Deletion of this acidic amino acid activation motif completely abolished CaMYB48 activation activity both in yeast and *in planta* (Fig. 4). The acidic amino acid domains have been reported to be responsible for the ability of MYBs to execute transcriptional activation processes^45^. The *Zea mays* C1 protein was the first MYB transcription factor identified in plants and is characterized as a transcriptional activator by numerous acidic residues at the C-terminus^46,47^. Recently, the *Medicago truncatula* MYB protein WP1 was elucidated to function as a transcriptional activator by directly regulating the expression of carotenoid biosynthetic genes through its C-terminal acidic activation motif^43^. These previous results revealed that the acidic region is essential for the activation activity of the MYB transcription factor. A BLASTP search and sequence analysis showed that the CaMYB48 acidic amino acid activation motif had no sequence similarity with any functionally characterized activation motifs. We speculate that convergent evolution of the MYB repeat domain ensures that the protein binds to the consensus DNA sequence, while divergent evolution of the activation motif enables the diversified functions of MYB transcription factors. However, the tested acidic amino acid activation motif contained 24 amino acid residues. We cannot rule out the possibility that shortening the tested amino acid residues would be sufficient for activation activity. Further shortening the amino acid residues and mutating the corresponding acidic amino acids enabled us to elucidate the activation mechanism.

In this study, through WGCNA, we identified a MEblack module related to capsaicinoid biosynthesis. The module provides candidate genes for the identification of new biosynthetic genes relevant to capsaicinoids. We elucidated that CaMYB48 functions as a transcriptional activator to regulate capsaicinoid biosynthesis. The identification of CaMYB48 provides a foundation for further research investigating the transcriptional regulatory networks for capsaicinoid biosynthesis in peppers and offers mechanistic insights into the evolution of plant secondary metabolism.

## Materials and methods

### Plant materials

The pepper (*C. annuum*) 59 inbred line is an elite line with high yield, high pungency, resistance to diverse pathogens and good heat, and drought tolerance. Thirty-five-day-old seedlings were transplanted into 25-cm plastic pots. The plants were grown in a greenhouse with a daily temperature of 25–27 °C, nighttime temperature of 18–20 °C, relative humidity of 65%, 16/8-h light/dark cycle, and light intensity of 6500 Lux. After pepper fruit development to the selected stages, the fruits were sampled. The fruits were dissected to separate the pericarp, seeds, and placenta. The samples were frozen in liquid nitrogen and stored in a −80 °C freezer.

### Identification of coexpression modules and visualization of gene expression

CM334 is a Mexican landrace that has consistently exhibited high levels of resistance to diverse pathogens. This landrace has been extensively used in hot pepper research and cultivar breeding, and its genome sequence was released in 2014^4^. The nonpungent cultivar ECW has a large deletion in *AT3a* that spans the region from the promoter to the first exon, leading to loss of *AT3a* (also known as *CS*) expression but without substantial changes in the expression of other genes in the biosynthetic pathway^4^. The 6421 inbred line was selected from a long-red-pepper landrace widely grown in Hunan Province, China. It is resistant to diverse pathogens and abiotic stress, and many F1 hybrids have been bred using 6421 as a female parent, which has been planted in many Chinese pepper-growing areas. The transcriptome data of pepper (*C. annuum*) cultivar CM334 (pungent), cultivar ECW (nonpungent) and inbred line 6421 (pungent) were retrieved from public databases^4,36^. These data include transcription data of genes from different tissues and fruits at different developmental stages. A gene coexpression network was built using the WGCNA package in R^33^. The networks were visualized using Cytoscape v.3.6.0^48^.

For high-throughput display of the expression levels of the assigned genes, heat maps were created. Related gene expression values were retrieved from public databases; the heat maps were plotted with the pheatmap package in R.

### RNA extraction

Quantitative real-time reverse transcription PCR (RT-PCR) analysis of gene expression in different tissues was performed. Roots, leaves, stems, and apical buds were collected from 30-day-old seedlings. Fully blossomed flowers were collected from 80-day-old plants. Seeds and pericarp were collected from 16-DPA fruit, and the placenta was collected at different fruit developmental stages. Total RNA was extracted from the samples using a HiPure HP Plant RNA Mini Kit (Magen, China). The RNA samples were used for further analysis as described below.

### Determination of Cap and DhCap contents

To determine the Cap and DhCap contents in the placenta of 59 inbred line pepper fruits from the indicated samples, the extraction and quantification of Cap and DhCap were performed as previously reported^5^. Briefly, ~0.1-g dry weight samples were extracted with 10 mL of a methanol/tetrahydrofuran (1:1) extract solution in a test tube at room temperature for 24 h. The Cap and DhCap standards (Sigma-Aldrich) or extracts were injected into an XSelect HSS C-18 SB column (4.6 × 250 mm, 5 μm, Waters Technologies, USA) and separated using 20% water (A) and 80% methanol (B) as mobile phases on a Waters Alliance Series HPLC system (Waters Technologies, USA). Detection was performed at 280 nm for Cap and DhCap.

### Sequence alignment and phylogenetic tree construction

To retrieve the MYB transcription factor sequences from other plant species, CaMYB48 was used as the bait gene to blast against the NCBI, Sol Genomics Network and TAIR databases. The MYB transcription factor sequences were aligned by CLUSTALX 2 with the default parameters, and the phylogenetic tree was constructed by MEGA 7.0 using the neighbor-joining method with 1000 bootstrap replicates^49^. The MYB transcription factor motifs displayed in this study were predicted using the online version of MEME.

### Quantitative real-time RT-PCR analysis

Total RNA was extracted from the tissues as described, and 1 μg of total RNA from each sample was subjected to reverse transcription using HiScript III RT SuperMix for qPCR (+gDNA wiper) eraser (Vazyme Biotech, China). AceQ Universal SYBR qPCR Master Mix (Vazyme Biotech, China) was applied for quantitative real-time PCR analysis. Analysis was performed on a LightCycler 480 Real-Time PCR System according to the manufacturer’s instructions (Roche, Switzerland), and the program was run as previously described^50^. The transcript level of the reference gene actin was used to quantify the relative transcript level of each target gene in each sample^51^. The values are means of three biological replicates. The primers used in this study are listed in Supplementary Table 3.

### Subcellular localization

The full-length coding sequence of *CaMYB48* was cloned into the pEAQ-GFP vector and fused with GFP under the control of the CaMV 35S promoter. The *Agrobacterium tumefaciens* strain GV3101 containing the corresponding constructs was infiltrated into young *N. benthamiana* leaves. After *N. benthamiana* leaves were inoculated with *Agrobacterium tumefaciens* for 3 days, the GFP signal was detected by confocal fluorescence microscopy (Carl Zeiss, Germany). The SV40 antigen (PKKKRKV) was used as a nuclear localization signal (NLS). To indicate the nuclei, the nucleotide sequence encoding the NLS was in frame with DsRed for nuclear targeting of the DsRed protein. The solution was infiltrated into *N. benthamiana* leaves. The primers used in this study are listed in Supplementary Table 3.

### Transcriptional activation analysis

A yeast transcriptional activation assay was performed as described in the manufacturer’s instructions for the Matchmaker Gold yeast two-hybrid system (Clontech, USA). Full-length *CaMYB48* or truncated *CaMYB48* was cloned into pGBKT7 to generate BDCaMYB48. The BDCaMYB48 or the BD empty vector was then transformed into the *Saccharomyces cerevisiae* strain Y2H Gold and incubated on SD/–Trp medium at 30 °C for 3 days. The positive clones were picked and diluted in 0.9% NaCl solution, and 10 µL of each dilution was inoculated onto SD/–Trp–His–Ade medium. After 3–5 days, the clones were stained with x-α-Gal (Clontech, USA). The primers used in this study are listed in Supplementary Table 3.

The transcriptional activation analysis of CaMYB48 *in planta* was carried out according to a previous study^5^. Full-length or truncated coding sequences of CaMYB48 were cloned into the pEAQ-BD vector and fused with the GAL4 DNA-binding domain under the control of the CaMV 35S promoter. The GAL-LUC reporter contained a firefly LUC gene driven by the minimal CaMV 35S promoter and five GAL4-binding elements. The *Renilla* LUC gene under the control of CaMV 35S on the same plasmid served as an internal control. The *Agrobacterium tumefaciens* strain GV3101 containing the effector or pEAQ-BD and reporter was coinfiltrated into young *N. benthamiana* leaves. Three days after infection, the firefly LUC and *Renilla* LUC activities were measured with a dual-LUC reporter assay system (Promega, USA) on a Varioskan™ LUX multimode microplate reader (Thermo Fisher Scientific, USA). The primers used are listed in Supplementary Table 3.

### Y1H assay

The full-length coding sequence *CaMYB48* was cloned into pGADT7 to generate prey AD-CaMYB48. The promoter fragments of *KasIa* and *AT3a* were ligated into the pAbAi vector to generate baits. The Y1H experiment was carried out according to the manufacturer’s protocol for the Matchmaker Gold Y1H library screening system (Clontech, USA). The primers used in this study are listed in Supplementary Table 3.

### VIGS analysis

*CaMYB48* coding sequence fragments that shared low similarity with other genes were cloned into pTRV2 to generate the silencing vector pTRV2-CaMYB48. The constructs included pTRV1, pTRV2, and pTRV2-CaMYB48 of *Agrobacterium tumefaciens* strain GV3101. *Agrobacterium tumefaciens* containing pTRV2-CaMYB48 or pTRV2 and pTVR1 vectors was coinfiltrated into 35-day-old seedlings of the 59 inbred line of pepper. The plants were grown in a greenhouse with a daily temperature of 24 °C, nighttime temperature of 18 °C, relative humidity of 70%, 16/8-h light/dark cycle, and light intensity of 6500 Lux. Total RNA was extracted from 16-DPA fruit placentas for expression analysis to evaluate silencing efficiency. The 45 PDA fruit placentas were used for Cap and DhCap content measurements. The primers used in this study are listed in Supplementary Table 3.

### Dual-LUC assay

The upstream start codons of *AT3a* (1843 bp) and *KasIa* (1917 bp) were PCR amplified from genomic DNA of the pepper inbred line 59 and cloned into pGreenII-0800 to serve as reporters. Within this vector, the *Renilla* gene under the control of a 35S promoter was used for the normalization of transfection efficiency. Full-length *CaMYB48* was PCR amplified from cDNA from the placenta of pepper inbred line 59 and cloned into the pEAQ vector under the CaMV 35S promoter to serve as an effector. The *Agrobacterium tumefaciens* strain GV3101 containing the *CaMYB48* effector and the corresponding reporters were coinfiltrated into young *N. benthamiana* leaves. After incubation for 3 days, the firefly LUC and *Renilla* LUC activities were measured with a dual-LUC reporter assay system (Promega, USA) on a Varioskan™ LUX (Thermo Fisher Scientific, USA). Activity is expressed as the ratio of firefly LUC activity to *Renilla* LUC activity. The primers used in this study are listed in Supplementary Table 3.

### Electrophoretic mobility shift assays (EMSAs)

To express recombinant protein, full-length *CaMYB48* was cloned into the pMAL-c2-x vector and fused in frame with the MBP tag. The constructs transformed into *E. coli* strain BL21 were grown in liquid medium to an OD at 600 nm of 0.4, treated with 1-mM IPTG to induce expression, and grown for 16 h at 16 °C with 130 rpm. The recombinant protein was purified with amylose beads according to the manufacturer’s instructions (NEB, USA). The probe DNA containing the wild-type cis-element and mutated cis-element probes was labeled using a Pierce Biotin 3′ End DNA Labeling Kit (Thermo Fisher Scientific, USA). EMSA was performed using a LightShift Chemiluminescent EMSA Kit (Thermo Fisher Scientific, USA) according to the manufacturer’s instructions. The primers used in this study are listed in Supplementary Table 3.

## Supplementary information

Supplementary Information Figures and Tables
