# Supplementary material for: Coexpression network analysis reveals an MYB transcriptional activator involved in capsaicinoid biosynthesis in hot peppers
Source: Hortic Res. 2020 Oct 1;7:162. doi: 10.1038/s41438-020-00381-2 (PMC7527512; doi:10.1038/s41438-020-00381-2)
Supplement: Supplementary file 1 — Supplementary Information Figures and Tables [file 41438_2020_381_MOESM1_ESM.docx]

**Coexpression network analysis identified an MYB transcriptional activator involved in capsaicinoid biosynthesis in hot peppers**

Running title: An MYB functions as a transcriptional activator involved in regulating capsaicinoid biosynthesis

Binmei Sun^1^, Xin Zhou^1,6^, Changming Chen^1^, Chengjie Chen^1^, Kunhao Chen^3,4^, Muxi Chen^3,4^, Shaoqun Liu^1^, Fanrong Cao^1^, Guoju Chen^1^, Bihao Cao^1^, Jianjun Lei^1,5*^ and Zhangsheng Zhu^1,2*^

1.Key Laboratory of Biology and Genetic Improvement of Horticultural Crops (South China), Ministry of Agriculture and Rural Affairs, College of Horticulture, South China Agricultural University, Guangzhou, 510642 China

2. Peking University-Southern University of Science and Technology Joint Institute of Plant and Food Sciences, Department of Biology, Southern University of Science and Technology, Shenzhen, 518055, China

3. Guangdong Helinong Seeds, CO.LTD, Shantou, Guangdong 515800, China

4. Guangdong Helinong Agricultural Research Institute, CO.LTD, Shantou, Guangdong, 515800, China

5. Henry School of Agricultural Science and Engineering, Shaoguan University, Guangdong, 512005, China

6. Jiangxi Agricultural Engineering College, Zhangshu, Jiangxi, 331200, China

Correspondence: zhuzhangsheng@stu.scau.edu.cn or zhuzs@sustech.edu.cn; jjlei@scau.edu.cn


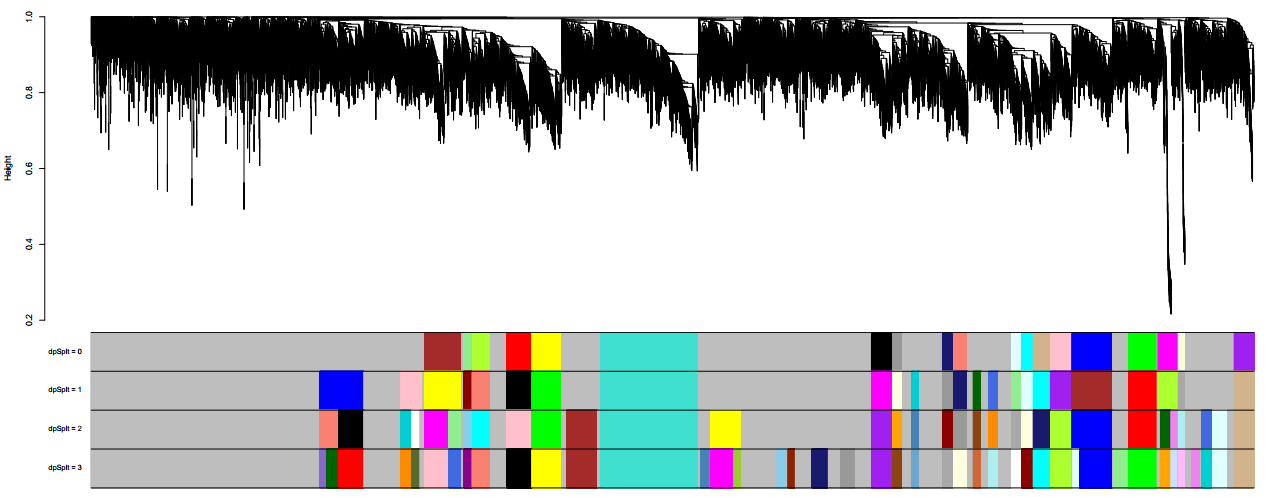


Supplementary Fig. 1 Hierarchical clustering tree. Hierarchical cluster tree showing coexpression modules identified by WGCNA. Each leaf in the tree is one gene. The major tree branches constitute 26 modules labeled by different colors.


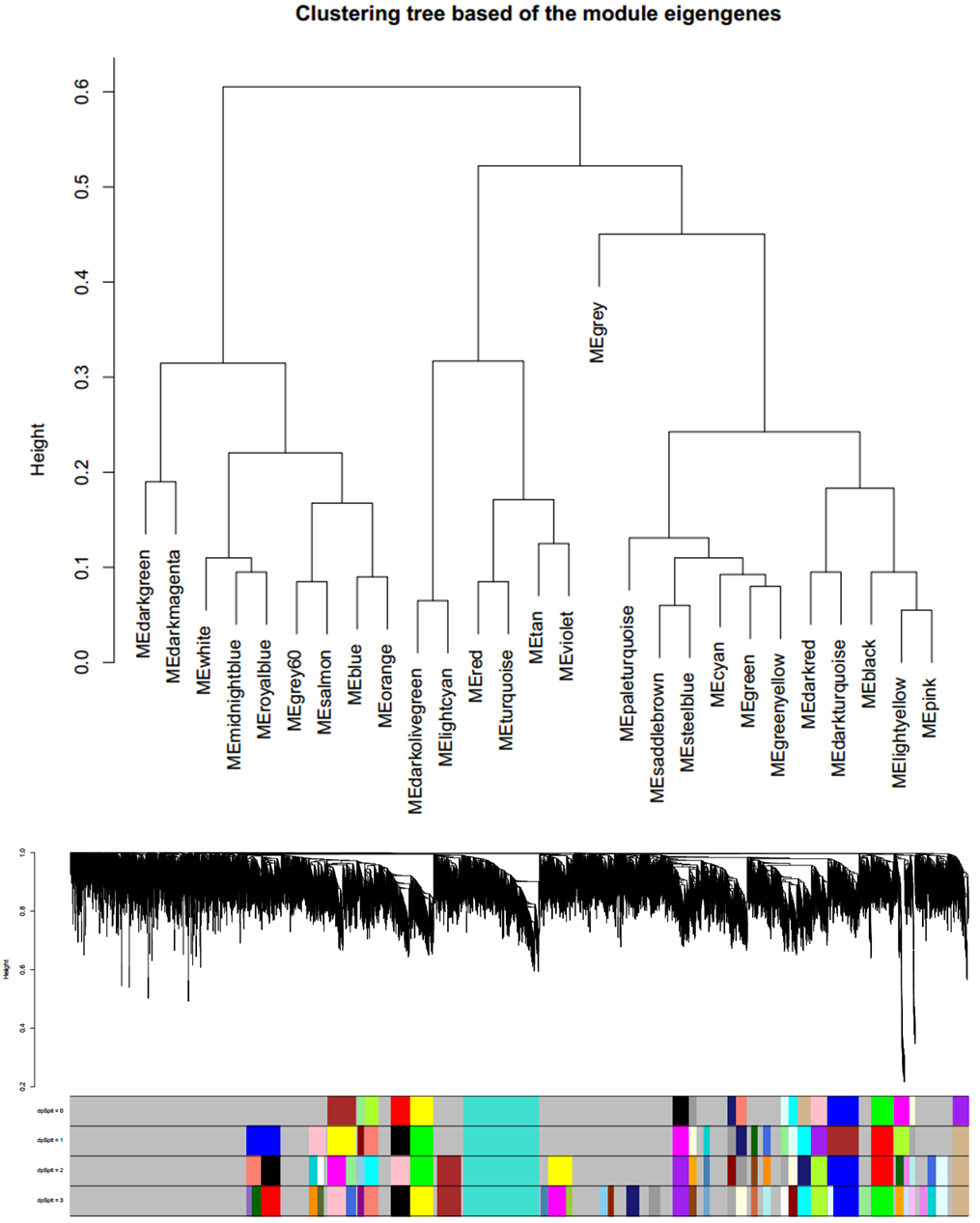


Supplementary Fig. 2 Cluster tree based of the module eigengenes.


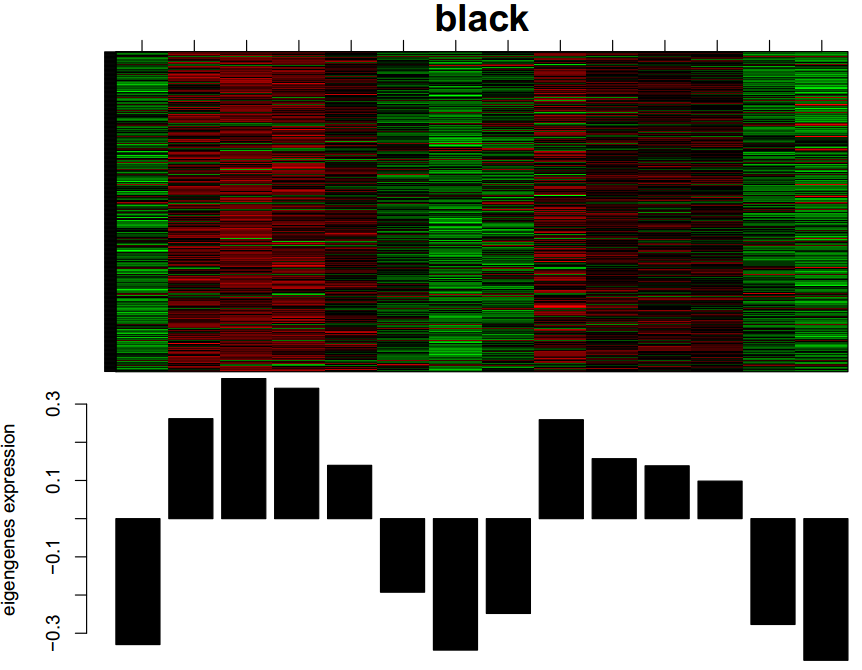


Supplementary Fig. 3 The expressions pattern of genes in MEblack module.

**Supplementary Table 1** The MEblack module CBGs

| GeneID | Description | kTotal | kWithin | kOut | kDiff | kME | kME-pvalue | Module Color | CM334 | | | | | | | ECW | | | | | | |
| --- | --- | --- | --- | --- | --- | --- | --- | --- | --- | --- | --- | --- | --- | --- | --- | --- | --- | --- | --- | --- | --- | --- |
|  |  |  |  |  |  |  |  |  | 6 PDA | 16 DPA | 25 DPA | 30 DPA | 33 DPA | 38 DPA | 48 DPA | 6 PDA | 16 DPA | 25 DPA | 30 DPA | 33 DPA | 38 DPA | 48 DPA |
| CA06g08940 | BCKDH | 31.085 | 28.961 | 2.124 | 26.836 | 0.987 | 7.83E-11 | black | 9.128 | 820.642 | 886.394 | 1023.308 | 194.185 | 27.44 | 23.069 | 20.396 | 844.447 | 302.067 | 209.452 | 170.871 | 15.032 | 8.749 |
| CA01g06170 | 4CL | 26.043 | 23.904 | 2.139 | 21.764 | 0.979 | 1.12E-09 | black | 2.949 | 151.934 | 118.092 | 113.435 | 47.31 | 9.485 | 6.017 | 4.714 | 76.98 | 45.09 | 46.771 | 23.673 | 3.136 | 2.744 |
| CA03g07640 | AMT | 41.097 | 32.464 | 8.633 | 23.83 | 0.968 | 1.42E-08 | black | 1.348 | 3436.423 | 2059.729 | 2021.653 | 135.365 | 3.472 | 1.668 | 28.93 | 2817.826 | 889.651 | 410.453 | 400.636 | 3.204 | 1.555 |
| CA01g25650 | KasIb | 37.311 | 32.084 | 5.227 | 26.857 | 0.962 | 3.92E-08 | black | 0.298 | 94.427 | 85.679 | 71.16 | 9.444 | 0.205 | 0.556 | 1.607 | 151.188 | 43.816 | 22.648 | 21.377 | 0.113 | 0.065 |
| CA02g18620 | AT3a | 32.176 | 26.676 | 5.5 | 21.175 | 0.946 | 3.05E-07 | black | 1.025 | 3686.118 | 808.431 | 61.239 | 2.64 | 0.733 | 0.77 | 0.316 | 1.429 | 0.412 | 0.383 | 0.498 | 0 | 0 |
| Acl | Acl | 31.126 | 22.375 | 4.923 | 7.171 | 0.946 | 3.01E-07 | black | 785.5671 | 7398.753 | 3606.431 | 5718.251 | 2665.992 | 827.5272 | 602.9807 | 590.8486 | 2803.116 | 1231.965 | 1151.569 | 1128.828 | 452.9235 | 523.2365 |
| CA01g00840 | KasIa | 33.498 | 23.842 | 9.656 | 14.186 | 0.937 | 7.84E-07 | black | 1.055 | 1495.969 | 1018.773 | 498.08 | 107.446 | 1.8 | 2.53 | 28.222 | 2597.999 | 876.656 | 500.62 | 445.294 | 7.181 | 2.455 |
| CA00g52190 | CCoAOMT | 15.203 | 6.481 | 8.722 | -2.241 | 0.89 | 2.00E-05 | black | 126.149 | 797.51 | 413.778 | 326.815 | 215.662 | 129.324 | 51.475 | 74.1 | 436.475 | 251.059 | 356.232 | 255.647 | 66.501 | 16.288 |
| CA00g41610 | KR | 5.878 | 5.087 | 0.79 | 4.297 | 0.83 | 0.000242285 | black | 100.249 | 682.134 | 414.4 | 394.951 | 200.876 | 133.555 | 111.459 | 109.973 | 487.876 | 152.621 | 125.155 | 122.487 | 84.851 | 86.427 |
| CA06g08600 | KasIIIa | 18.079 | 9.388 | 8.692 | 0.696 | 0.804 | 0.000525073 | black | 0.105 | 36.201 | 15.6 | 3.914 | 0.522 | 0 | 0 | 2.218 | 47.39 | 25.873 | 16.944 | 13.171 | 0.076 | 0 |
| CA00g02500 | DH | 5.193 | 3.098 | 2.095 | 1.002 | 0.779 | 0.001036027 | black | 245.88 | 615.527 | 272.409 | 272.272 | 151.214 | 103.08 | 116.197 | 223.573 | 227.878 | 129.531 | 156.008 | 171.305 | 104.005 | 117.579 |
| CA00g33160 | MACT | 0.613 | 0.48 | 0.133 | 0.348 | 0.711 | 0.004376262 | black | 131.619 | 543.296 | 229.868 | 377.774 | 242.156 | 225.18 | 176.881 | 176.357 | 399.311 | 183.719 | 258.048 | 312.62 | 199.307 | 208.167 |
| CA00g30270 | FatA | 2.242 | 1.214 | 1.028 | 0.186 | 0.699 | 0.005377819 | black | 17.198 | 252.017 | 459.18 | 179.627 | 88.438 | 90.141 | 115.257 | 30.988 | 553.1 | 159.093 | 87.685 | 88.57 | 124.735 | 83.571 |
| CA04g13860 | BCAT | 3.459 | 1.148 | 2.311 | -1.162 | 0.679 | 0.007519682 | black | 0 | 72.484 | 189.703 | 72.903 | 8.647 | 28.782 | 21.508 | 6.316 | 496.74 | 124.851 | 52.349 | 39.433 | 36.437 | 22.836 |

**Supplementary Table 2** The MEblack module transcription factors

| GeneID | Description | kTotal | kWithin | kOut | kDiff | kME | kME-pvalue | Module Color | CM334 | | | | | | | ECW | | | | | | |
| --- | --- | --- | --- | --- | --- | --- | --- | --- | --- | --- | --- | --- | --- | --- | --- | --- | --- | --- | --- | --- | --- | --- |
|  |  |  |  |  |  |  |  |  | 6 PDA | 16 DPA | 25 DPA | 30 DPA | 33 DPA | 38 DPA | 48 DPA | 6 PDA | 16 DPA | 25 DPA | 30 DPA | 33 DPA | 38 DPA | 48 DPA |
| CA11g12490 | R2R3-myb transcription factor, putative | 25.31663 | 13.34629 | 11.97034 | 1.37595 | 0.952797 | 1.44E-07 | black | 10.391 | 64.212 | 97.197 | 51.046 | 34.633 | 17.936 | 2.927 | 8.768 | 37.857 | 46.02 | 35.937 | 31.445 | 9.059 | 4.263 |
| CA00g60490 | WRKY transcription factor, putative | 16.64905 | 15.51704 | 1.132009 | 14.38503 | 0.949851 | 2.06E-07 | black | 0.093 | 10.689 | 19.575 | 24.776 | 4.663 | 3.22 | 0.545 | 0.868 | 34.719 | 11.66 | 6.296 | 4.87 | 0.828 | 0.393 |
| CA00g38550 | AP2 domain-containing transcription factor | 41.59306 | 29.77479 | 11.81828 | 17.95651 | 0.986671 | 2.35E-07 | black | 0.561 | 66.784 | 88.292 | 124.63 | 33.902 | 1.411 | 0.102 | 3.712 | 82.057 | 38.353 | 34.11 | 29.658 | 0.225 | 0 |
| CA00g51500 | TCP transcription factor 4 | 11.18795 | 10.18318 | 1.004778 | 9.178399 | 0.936843 | 7.99E-07 | black | 0 | 4.592 | 10.535 | 14.981 | 4.725 | 1.487 | 0.871 | 0.506 | 10.482 | 5.972 | 2.42 | 1.732 | 0.108 | 0.061 |
| CA06g01960 | MYB transcription factor | 20.7763 | 10.46865 | 10.30765 | 0.161002 | 0.917774 | 3.73E-06 | black | 1.429 | 12.234 | 53.145 | 25.984 | 24.852 | 1.519 | 0 | 0.461 | 4.057 | 10.235 | 6.799 | 6.081 | 0.166 | 0 |
| CA08g08670 | Transcription factor | 12.34052 | 7.397635 | 4.94288 | 2.454755 | 0.89935 | 1.20E-05 | black | 25.182 | 73.781 | 234.851 | 147.808 | 174.923 | 65.867 | 26.145 | 58.139 | 166.44 | 164.855 | 124.476 | 124.251 | 52.388 | 18.558 |
| CA00g86400 | NAC domain protein, IPR003441 | 17.1275 | 8.40864 | 8.718861 | -0.31022 | 0.895336 | 1.51E-05 | black | 39.833 | 69.589 | 74.128 | 87.807 | 59.343 | 42.463 | 32.515 | 39.711 | 52.179 | 46.067 | 48.416 | 44.376 | 36.595 | 32.325 |
| CA07g19720 | NAC domain protein IPR003441 | 5.938626 | 3.214729 | 2.723897 | 0.490832 | 0.859591 | 8.12E-05 | black | 1.521 | 1.91 | 7.325 | 6.92 | 4.446 | 1.12 | 0.701 | 1.153 | 3.276 | 3.207 | 1.454 | 3.186 | 0.291 | 0.529 |
| CA00g27590 | NAC transcription factor | 6.147812 | 4.464615 | 1.683197 | 2.781417 | 0.845105 | 0.000141586 | black | 2.046 | 20.288 | 24.388 | 30.523 | 17.181 | 3.581 | 5.192 | 1.254 | 4.52 | 17.503 | 23.324 | 9.836 | 1.66 | 3.188 |
| CA02g05210 | BHLH transcription factor | 22.16994 | 7.000912 | 15.16903 | -8.16812 | 0.844609 | 0.000144164 | black | 2.388 | 9.907 | 17.397 | 4.53 | 5.59 | 0.217 | 0 | 2.009 | 7.619 | 3.621 | 2.152 | 1.366 | 0 | 0 |
| CA00g59070 | BZIP transcription factor protein | 5.552475 | 2.461238 | 3.091237 | -0.63 | 0.843089 | 0.000152309 | black | 224.485 | 328.315 | 425.382 | 416.68 | 355.664 | 292.104 | 223.707 | 298.459 | 396.027 | 340.737 | 371.321 | 407.385 | 265.855 | 324.914 |
| CA02g09260 | TCP domain class transcription factor | 3.890662 | 2.507655 | 1.383007 | 1.124648 | 0.821427 | 0.000314868 | black | 3.318 | 5.829 | 11.503 | 7.942 | 7.155 | 3.664 | 5.987 | 6.317 | 10.41 | 7.928 | 7.812 | 6.686 | 3.014 | 2.293 |
| CA00g26680 | GATA transcription factor, putative | 16.62809 | 5.886946 | 10.74115 | -4.8542 | 0.805313 | 0.000509432 | black | 8.844 | 17.172 | 15.935 | 13.593 | 8.713 | 6.641 | 5.471 | 10.062 | 17.266 | 13.67 | 9.983 | 8.431 | 3.682 | 6.791 |
| CA06g12860 | Transcription factor, putative | 6.80423 | 3.20664 | 3.59759 | -0.39095 | 0.803301 | 0.000539319 | black | 70.781 | 110.942 | 196.918 | 200.582 | 145.415 | 107.439 | 94.837 | 89.126 | 102.394 | 106.384 | 119.392 | 110.167 | 92.63 | 74.993 |
| CA06g12670 | Transcription factor, putative | 3.416674 | 2.478976 | 0.937698 | 1.541277 | 0.794717 | 0.000683107 | black | 10.576 | 25.848 | 46.525 | 40.491 | 26.73 | 21.319 | 25.478 | 12.08 | 19.153 | 33.189 | 36.549 | 34.359 | 8.727 | 8.492 |
| CA02g24640 | TCP transcription factor 5 | 3.306769 | 1.404308 | 1.90246 | -0.49815 | 0.747019 | 0.002138874 | black | 0.315 | 1.719 | 13.483 | 10.115 | 4.181 | 1.593 | 2.861 | 5.074 | 28.505 | 11.822 | 4.734 | 3.889 | 0.134 | 0.439 |
| CA02g13080 | WRKY transcription factor 6 | 12.86111 | 3.475498 | 9.38561 | -5.91011 | 0.735032 | 0.002744232 | black | 1.98 | 8.046 | 20.984 | 29.433 | 18.922 | 7.413 | 3.086 | 1.224 | 2.503 | 5.312 | 6.143 | 6.233 | 3.579 | 1.354 |
| CA00g40340 | WRKY1 | 0.956609 | 0.575371 | 0.381239 | 0.194132 | 0.689285 | 0.006390605 | black | 67.111 | 147.829 | 157.332 | 197.258 | 134.788 | 60.552 | 21.39 | 44.502 | 296.621 | 82.87 | 69.816 | 91.125 | 74.462 | 136.274 |
| CA00g27760 | NAC transcription factor | 1.303338 | 0.795279 | 0.508058 | 0.287221 | 0.66128 | 0.010014658 | black | 2.825 | 12.293 | 12.847 | 13.638 | 12.858 | 2.757 | 6.918 | 1.187 | 4.379 | 22.287 | 27.506 | 13.316 | 4.717 | 5.804 |
| CA06g11620 | MYB transcription factor MYB34 | 1.323423 | 0.328895 | 0.994528 | -0.66563 | 0.623173 | 0.017271772 | black | 12.321 | 8.686 | 19.162 | 21.626 | 12.464 | 7.445 | 9.627 | 14.172 | 11.919 | 15.428 | 18.186 | 15.717 | 8.178 | 8.347 |

**Supplementary Table 3** Sequence of primers

| Name | Forward | Reverse |
| --- | --- | --- |
| qPAL | AATCAATCCCTCCACCTCTT | TGACCCATCCCTACCATAAC |
| qC4H | CATTGGAGGAAGATGAGGAGGA | CTAAGCTTTACGAAAGGGGGAT |
| qCCoAMTa | CTTGCTATTCAATCTTTTCTCAC | CCTCCCACATCTATTATCTCTTT |
| qAMT | AAGACTCAAGGTAAATGGTGGA | TAATTGGGGAGATAAGGGGAAC |
| qKasIa | TTATAGGTTCTTTCTCCCCA | GCCATTACATTCTTATGTTTGTC |
| qBCAT | GAAATAATTACCCACAGGGCAGG | GTTTAGAAGAGAGGATGGACGAC |
| qBCEDH | TGCTTCTGCTGCTGCTCTATCTC | CTGCCTTTCCCTTTATCATCCTT |
| qFatA | GAAGGCGTCTTGTGTCCTGGTT | TGCTTGGAGTGATGTTGTTGAA |
| qAcl | TGGCTTCTATTACTGCATCTTC | CACACTTTGTCAACTGTCTCTG |
| qAT3a | AAACCTTCCTCTCTCACCCCCTCT | AGCATTGTCCTTCAACTTTCCAGC |
| qCaMYB48 | GTTTGAAAAGAACAGGAAAGAG | AAATGATGAAGATGGAGAGATAGA |
| qActin2  Reference gene | AATCAATCCCTCCACCTCTTCACTC | CATCACCAGCAAATCCAGCCTT |
| BDCaMYB48  1-222aa | ATGGCCATGGAGGCCGAATTCATGGCGCAAGAGGAAATAATGA | CCGCTGCAGGTCGACGGATCCTTATCGAAAGCTACTCCAATCAAAA |
| BDCaMYB48  1-115aa | ATGGCCATGGAGGCCGAATTCATGGCGCAAGAGGAAATAATGA | CCGCTGCAGGTCGACGGATCCTTACCTTTGTTCTTGAGCTTTCTT |
| BDCaMYB48  116-222aa | ATGGCCATGGAGGCCGAATTCAAAAAGTCTTCTATCTCTCCATCT | CCGCTGCAGGTCGACGGATCCTTATCGAAAGCTACTCCAATCAAAA |
| BDCaMYB48  168-222aa | ATGGCCATGGAGGCCGAATTCGGAGAAAGTATGAAAATTTACTCC | CCGCTGCAGGTCGACGGATCCTTATCGAAAGCTACTCCAATCAAAA |
| BDCaMYB48  116-167aa | ATGGCCATGGAGGCCGAATTCAAAAAGTCTTCTATCTCTCCATCT | CCGCTGCAGGTCGACGGATCCACCTTGTTCTTGATCACTAGATTTCAT |
| BDCaMYB48  168-191aa | ATGGCCATGGAGGCCGAATTCGGAGAAAGTATGAAAATTTACTCC | CCGCTGCAGGTCGACGGATCCTTCTTCATTTTCTTCTAATAATTCAATAT |
| BDCaMYB48  192-222aa | ATGGCCATGGAGGCCGAATTCACAACGAATAAACCAATTAT | CCGCTGCAGGTCGACGGATCCTTATCGAAAGCTACTCCAATCAAAA |
| pEAQ-BDMYB48  1-222aa | TCGCCGACCGGTAGGCCTATGGCGCAAGAGGAAATA | AACCAGAGTTAAAGGCCTTTATCGAAAGCTACTCCAATC |
| pEAQ-BDMYB48  1-115aa | TCGCCGACCGGTAGGCCTATGGCGCAAGAGGAAATA | AACCAGAGTTAAAGGCCTTTATCGAAAGCTACTCCAATC |
| pEAQ-BDCaMYB48  116-222aa | TCGCCGACCGGTAGGCCTAAAAAGTCTTCTATCTCTCCATCT | AACCAGAGTTAAAGGCCTTTATCGAAAGCTACTCCAATC |
| CaMYB48-GFP | GCCCTTGCTCACCATACCGGTTCGAAAGCTACTCCAATC | GCCCTTGCTCACCATACCGGTCATTTGTATCAACGTTTGTAAATTC |
| pTRV2-CaMYB48 | CGGAATTCCGAAACAGATGGTCAAGAAT | CGGGATCCCGGATTTCATTTCTCCTTCA |
| pEAQ-CaMYB48 | CTGCCCAAATTCGCGACCGGTATGGCGCAAGAGGAAATA | ACCAGAGTTAAAGGCCTCGAGTTATCGAAAGCTACTCCAATC |
| CaMYB48-MBP | CGGGATCC ATGGCGCAAGAGGAAATAATG | CGGAATTC TTATCGAAAGCTACTCCAATCA |
| ProAT3a | CTATAGGGCGAATTGGGTACCACTCTTCAATTTTCTTCCTTTTTGAGA | TGTTTTTGGCGTCTTCCATGGAATTAATTTAACAAATAAATATATGCTGCTG |
| ProKasIa | CTATAGGGCGAATTGGGTACCGATGTCTTTGATGTTTCCTATCCGA | TGTTTTTGGCGTCTTCCATGGGGCCCTGATTCTTCCACATTT |
| ADCaMYB48 | GCCATGGAGGCCAGTGAATTCATGGCGCAAGAGGAAATA | CAGCTCGAGCTCGATGGATCCTTATCGAAAGCTACTCCAATC |
| pAbAi-AT3a | GGGGTACC GCCAAAATAACTCTTCAAACA | CCCAAGCTT AGCTTAGTGGGGAATAATTG |
| pAbAi-KasIa | GGGGTACC AGAGAAGATAGAAATGCACTAGTAG | CCCAAGCTT GGAAAACACTCTTCATATCTTC |
| ProKasI-LUC | CTATAGGGCGAATTGGGTACCGATGTCTTTGATGTTTCCTATCCGA | TGTTTTTGGCGTCTTCCATGGGGCCCTGATTCTTCCACATTT |
| ProAT3-LUC | CTATAGGGCGAATTGGGTACCCACCTCGAAAGATATGACACGGAC | TGTTTTTGGCGTCTTCCATGGAATTAATTTAACAAATAAATATATGCTGCTGG |
| Wild type probe | AATTGGAGGGTGTTAGGTGTATTATATTAAATTTGTAAAGTT | AACTTTACAAATTTAATATAATACACCTAACACCCTCCAATT |
| Mutated probe | AATTGGAGGGTATTAAATAGATTATATTAAATTTGTAAAGTT | AACTTTACAAATTTAATATAATCTATTTAATACCCTCCAATT |
